# Molecular and serological evidence for the circulation of the tick symbiont *Midichloria* (Rickettsiales: Midichloriaceae) in different mammalian species

**DOI:** 10.1186/1756-3305-6-350

**Published:** 2013-12-12

**Authors:** Chiara Bazzocchi, Mara Mariconti, Davide Sassera, Laura Rinaldi, Elena Martin, Giuseppe Cringoli, Sandra Urbanelli, Claudio Genchi, Claudio Bandi, Sara Epis

**Affiliations:** 1 Dipartimento di Scienze Veterinarie e Sanità Pubblica, Università degli Studi di Milano, Milano, Italy; 2 Fondazione IRCCS Policlinico San Matteo, Pavia, Italy; 3 Dipartimento di Patologia e Sanità Animale, Università degli Studi di Napoli Federico II, Napoli, Italy; 4 Dipartimento di Biologia Ambientale, Università di Roma La Sapienza, Roma, Italy

**Keywords:** *Midichloria*, Ticks, Mammalian hosts, Emerging infections, Vector-borne diseases

## Abstract

**Background:**

The Midichloriaceae is a novel family of the order Rickettsiales, that encompasses intracellular bacteria associated with hard ticks (Ixodidae) and other arthropods. The most intensively investigated member of this family is *Midichloria mitochondrii*, a symbiotic bacterium of the sheep tick *Ixodes ricinus*, characterized by the capacity of multiplying inside the mitochondria. A recent study suggested that these bacteria might be inoculated into the human host during the tick bite. The purpose of this study was to determine the potential infectivity of *Midichloria* bacteria for non-human animals exposed to the risk of tick bite.

**Methods:**

Blood from horses, cattle, sheep and dogs exposed to the risk of tick bite was included in this study. DNAs were extracted, and amplified using 16S ribosomal RNA primers conserved in the *Midichloria* genus. Furthermore, sera from dogs exposed to the risk of tick bite were analyzed in order to evaluate the presence of antibodies against the recombinant flagellar protein (rFliD) from *M. mitochondrii* using an ELISA test.

**Results:**

Here we present two lines of evidence that support the possibility that bacteria from the genus *Midichloria* are inoculated into vertebrate hosts during a tick bite: (i) a direct evidence, i.e. the detection of circulating DNA from bacteria related with *M. mitochondrii*, in the blood of vertebrates exposed to tick parasitism; (ii) a further indirect evidence, i.e. the presence of antibodies against an antigen from *M. mitochondrii* in dogs exposed to the risk of tick bite. It is interesting to note that variability was detected in the *Midichloria* gene sequences recovered from positive animals, and that some of these sequences were identical to those generated from tick-associated *Midichloria*.

**Conclusions:**

Based on the results, and on the overall information so far published on the genus *Midichloria*, we suggest that these bacteria are likely to represent a novel group of vector-borne agents, with the potential of infecting mammalian hosts. Whether inoculation of *Midichloria* bacteria could cause a true infection and pathological alteration in mammalian hosts is still to be determined. Surely, results emphasize the relevance of *Midichloria* bacteria in investigations on tick immunology and tick-bite markers.

## Background

Ticks are responsible for the transmission of a number of bacterial, protozoan and viral diseases to humans and animals. In addition to well-established pathogenic bacteria, ticks frequently harbour microorganisms whose pathogenic role to vertebrates is still to be determined; some of these have been regarded as symbionts, capable of providing the host tick with some fitness advantage [1,2]. A recently described family of the Rickettsiales, *Candidatus* Midichloriaceae (hereafter Midichloriaceae), encompasses a wide group of intracellular bacteria associated with ticks [3,4]. The most intensively investigated member of the family Midichloriaceae is *Candidatus* Midichloria mitochondrii (hereafter *M. mitochondrii*), an intracellular bacterial symbiont that is widespread in the hard tick *Ixodes ricinus*[5,6], the main vector of Lyme disease in Europe. *M. mitochondrii* is present both in the cell cytoplasm and within the inter-membrane space of mitochondria in the ovary of the host tick [7,8]. Other ticks that have been shown to harbour bacteria attributable to the genus *Midichloria* include members of the genera *Ixodes*, *Rhipicephalus*, *Amblyomma*, *Hyalomma* and *Dermacentor*[3,9,10].

Recently, *M. mitochondrii* has been detected in the salivary glands of *I. ricinus*, and human patients parasitized by this tick have been shown to be seropositive toward an antigenic protein from this bacterium [11]. *M. mitochondrii* can thus be regarded not only as a symbiont of *I. ricinus,* but also as a potential infectious agent or, at least, as a package of antigens that ticks likely inject into the vertebrate hosts during the blood meal. The fate of *M. mitochondrii* in the vertebrate after the tick bite is indeed still unexplored, but there is circumstantial evidence for the presence of circulating DNA from *M. mitochondrii* in roe deer (as revealed during a screening for *Anaplasma phagocytophilum* in Denmark [12]). In addition, DNA from another bacterium of the family Midichloriaceae (known as ‘Montezuma’) was detected in human patients parasitized by *Ixodes persulcatus*[13]. Despite this circumstantial evidence, no systematic studies have so far been focused on the specific goal of determining whether DNA from bacteria of the genus *Midichloria* can be detected in blood samples from animals exposed to tick parasitism. In addition, no evidence has so far been published for the presence of anti-*Midichloria* antibodies in the sera from animals other than *Homo sapiens*. The goals of this work were thus to determine: i) whether circulating DNA from *Midichloria* bacteria is detectable in animals exposed to the risk of tick bite; ii) whether dogs exposed to the risk of tick parasitism are seropositive for anti-*Midichloria* antibodies.

## Methods

### Sample collection

One hundred fifty-six whole blood samples from different mammalian hosts exposed to the risk of tick bite were included in the study: 46 horses, 13 cattle and 11 sheep from non-intensive breeding farms, in which animals are allowed to graze out of the stable for 3–6 months during the year, in the regions Lombardia and Lazio, Italy; 62 dogs, form a kennel in Pantelleria Island, Italy; 4 dogs from a kennel in the Molise region, Italy; 20 dogs from two kennels in the Campania region, Italy (Table 1). In order to examine animals at low risk of tick bite, blood from 30 cattle from an intensive dairy farm and from 20 experimental dogs that had been collected in the context of a previous study [14], were included in the analyses. DNA from blood samples was extracted by using the QIAamp DNA blood mini kit (Qiagen, Hilden, Germany) according to the manufacturer’s instructions, eluted into 100 μl of sterile water and stored at −20°C until use. For serological analysis, a total of 218 dog sera were collected from 16 kennels (hereafter indicated as K1-K16) located in southern Italy (Table 2) and stored at −20°C until use. Sera from the above 20 experimental dogs were used as control samples also for serology.

**Table 1:** **Animals screened for the presence of circulating *Midichloria *** **DNA**

**Mammalian host**	**Region or origin**	**Sample size**	**No. of positive (%)**
Dog (Kp^a^)	Sicilia	62	18 (29%)
Dog (K1^b^)	Molise	4	1 (25%)
Dog (K3^b^)	Campania	7	1 (14%)
Dog (K14^b^)	Campania	13	0 (0%)
Horse (NI^c^)	Lazio	46	5 (11%)
Sheep (NI^c^)	Lombardia	11	1 (9%)
Cattle (NI^c^)	Lombardia	13	0 (0%)
Cattle (I^d^)	Lombardia	30	0 (0%)
Dog (control group)	Experimental animals^e^	20	0 (0%)

^a^dogs from a kennel in the Pantelleria Island (Kp), Sicilia; ^b^kennel code as indicated in Table 2; ^c^animals from non-intensive (NI) breeding farms; ^d^animals from an intensive (I) breeding farm; ^e^animals from a previous experimental study [14].

**Table 2:** **Dogs screened for the presence of anti-*Midichloria *** **antibodies**

**Kennel code or group**	**County or origin**	**No. of dogs**	**Positivity**^a^	**OD average ± SD (OD min-OD max)**
K1	Campobasso	15	86.6	0.46 ± 0.2 (0.22-0.8)
K2	Salerno	16	31.25	0.25 ± 0.07 (0.18-0.36)
K3	Avellino	19	73.68	0.33 ± 0.1 (0.2-0.53)
K4	Avellino	9	22.2	0.27 ± 0.16 (0.17-0.6)
K5	Avellino	9	55.5	0.27 ± 0.09 (0.19-0.48)
K6	Avellino	13	61.53	0.27 ± 0.08 (0.15-0.42)
K7	Napoli	12	8.3	0.14 ± 0.08 (0.1-0.32)
K8	Caserta	13	23	0.14 ± 0.12 (0.07-0.38)
K9	Avellino	15	20	0.20 ± 0.09 (0.09-0.39)
K10	Caserta	19	5.26	0.14 ± 0.06 (0.09-0.35)
K11	Napoli	16	12.5	0.16 ± 0.05 (0.08-0.27)
K12	Avellino	12	8.3	0.16 ± 0.08 (0.1-0.4)
K13	Napoli	16	0	0.12 ± 0.03 (0.09-0.17)
K14	Avellino	13	0	0.15 ± 0.04 (0.1-0.24)
K15	Salerno	16	0	0.13 ± 0.2 (0.05-0.1)
K16	Napoli	5	0	0.12 ± 0.06 (0.08-0.22)
Control	Exp. animals ^b^	20	5	0.13 ± 0.05 (0.07-0.28)

^a^percentage of positive animals, based on the cut-off determined on the basis of the results from control dogs (see Methods); ^b^animals from a previous experimental study [14].

### PCR analysis

**Figure 1:**
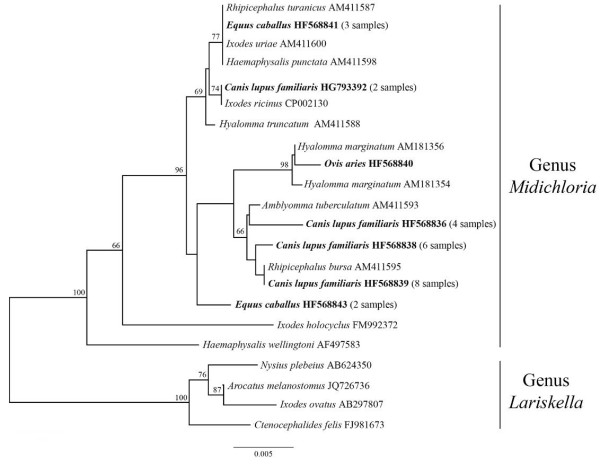
**Phylogenetic tree of *Midichloria *spp. 16S rDNA gene sequences.** Example of a phylogenetic tree showing the positioning of the *Midichloria* spp. 16S rDNA gene sequences generated from mammalian hosts (bold) relative to those generated in previous studies from ticks. In addition to sequences attributable to the genus *Midichloria*, other from the genus *Lariskella* have been included as outgroups. Names at the terminal nodes are those of the host organisms (mammalians, ticks, and other arthropod species in the case of the genus *Lariskella*). The tree was built using the Neighbor-joining method after Kimura two-parameters corrections, with the insertions/deletions not taken into account. Numbers at the nodes are the bootstrap confidence values; the scale bar indicates the number of substitutions per nucleotide. Accession numbers of the sequences are indicated at the terminal nodes. When multiple identical sequences were obtained from blood specimens of the same mammalian species, only one sequence was deposited in the data bases; in these cases, the number of specimens displaying identical sequences is given in parenthesis.

DNAs extracted from blood samples were analyzed for the presence of circulating DNA from *M. mitochondrii* or related bacteria, using a previously described PCR protocol, with primers designed on the gene coding for the 16S ribosomal RNA (16S rDNA) and targeted on portions of this gene that are conserved among representatives of the genus *Midichloria*[3,9]. All of the DNAs from blood samples were also examined using universal mammalian PCR primers, targeted on the 12S rRNA gene [15]. PCR products obtained with *Midichloria* primers were sequenced using ABI technology, and compared with the databases using BLAST (National Center for Biotechnology Information, Bethesda, Md). Seven of the obtained 16S rDNA sequences were deposited in the data bases (see Figure 1 for the accession numbers), with only one sequence deposited from each host species where the sequences from that host were identical.

### Phylogenetic reconstruction

The 16S rDNA sequences generated were aligned with the corresponding sequences from Midichloriaceae bacteria, including *Rickettsia rickettsii* as an outgroup. In addition, a sub-alignment was generated including sequences representative of the Midichloriaceae genera *Midichloria* and *Lariskella*. The alignment was generated using MUSCLE [16] and manually checked. Phylogenetic analysis were effected using the Neighbor joining method after Kimura 2-parameter correction or Jukes and Cantor correction, after either including or excluding the insertion/deletions, or using maximum likelihood with GTR substitution model. Phylogenetic analyses were performed using SeaView [17], estimating the reliability of the nodes after 100 bootstrap replicates.

### ELISA analysis

The recombinant flagellar protein FliD of *M. mithocondrii* (rFliD) was produced in *Escherichia coli* and purified as described in Mariconti *et al*. [18]. Wells of ELISA flat-bottom plates were coated with 0.1 μg/well of rFliD. Sera were analyzed in duplicate at a dilution of 1:100 and the anti IgG HRP-conjugated antibody was employed at 1:5000. The optical density (O.D.) was measured at a wavelength of 492 nm. The cut-off was established at an O.D. of 0.25, which is the mean O.D. of the 20 control sera plus three times their standard deviation. Samples with O.D. lower than 0.25 were classified as negative and samples with O.D. greater than or equal to 0.25 were classified as positive.

### Ethical statement

The study was approved from the National Ethics Committee of the involved institutes.

## Results and discussion

This work presents the first screening aimed at detecting DNA from tick-associated Midichloriaceae bacteria in mammalians. A total of 156 blood samples derived from horses, cattle, sheep and dogs at risk of tick bite were analyzed by PCR, using a previously described PCR protocol targeted on the 16S rDNA gene of *M. mitochondrii* and related bacteria [9]. Blood samples from 30 cattle and 20 dogs that are regarded as having no or limited risk of tick bite were also included. Details on the different groups of animals examined are listed in Table 1.

The quality of the DNAs extracted was checked by PCR using universal mammalian primers targeted on the mitochondrial 12S rRNA gene that led to positive amplification from all samples. *Midichloria*-targeted primers led to positive amplification from 26 of the animals at risk of tick bite, and from none of the control animals (Table 1). The animal species that presented the higher prevalence of positive animals was the dog, where 20 out of 86 animals were positive. Eighteen of these dogs were hosted in a kennel in the Pantelleria island (part of the Sicily region), where a dense population of the hard tick *Rhipicephalus bursa* has been recorded (Epis S., personal observation), while two dogs where hosted in two kennels in the Molise and in the Campania regions (Italy). The PCR products obtained from all *Midichloria*-positive animals (five horses, one sheep, twenty dogs) were recovered from the gel, purified and sequenced by ABI technology; the obtained sequences were manually corrected, compared with the databases using Blast, and included into different alignments with homologous sequences, for phylogenetic analyses. All of the sequences generated gave the best scores toward 16S rDNA sequences from Midichloriaceae bacteria. Phylogenetic analyses further confirmed that the sequenced PCR products derived from bacteria closely related with *M. mitochondrii*. In particular, in phylogenetic analyses including all of the sequences available from Midichloriaceae bacteria, the sequences here obtained from mammalians were placed into cluster 2 of this family (result not shown), which corresponds to the genus *Midichloria*[3]. Figure 1 presents an example of a phylogenetic tree obtained including representatives from the two main genera of the Midichloriaceae, *Midichloria* and *Lariskella*: the gene fragments generated from the blood samples here examined cluster with those of *Midichloria* bacteria previously detected in ticks (trees generated using different settings/algorithms presented the same placement for *Midichloria* sequences derived from blood samples). It has already been shown that *Midichloria* bacteria harbored by different tick species are variable at the level of the 16S rDNA [9].

The tree in Figure 1 shows that the novel sequences from mammalians are in some cases identical to those obtained from ticks: sequences from three horses to those obtained from three different tick species (*Ixodes uriae*, *Haemaphysalis punctata* and *Rhipicephalus turanicus*; sequences published in ref. [9]); sequences from eight dogs from the Pantelleria island to those obtained from *R. bursa*[9]; sequences from two dogs from the Molise and Campania regions to those obtained from *I. ricinus*[6]. Other 16S rDNA gene sequences obtained from the examined mammalians differ from those so far generated from ticks (showing from 2 to 5 nucleotide substitutions from the closest 16S rDNA *Midichloria* sequence from ticks). This result is not surprising, considering that only a minimal proportion of the ticks present in Italy have been screened for *Midichloria* bacteria [9]; the overall molecular diversity of these bacteria is thus still to be determined. In summary, we can affirm that DNA from bacteria attributable to the genus *Midichloria* can be detected in the blood of different animal species, and, in at least a few of the cases here examined, we can reasonably hypothesize that the origin of the detected DNA could be traced to ticks. For example, *R. bursa*, *I. ricinus* and *R. turanicus* could be involved in the transmission of *Midichloria* bacteria (or their DNA) to dogs and horses, also considering the host spectrum of these tick species and/or their distribution in the areas where the blood samples were collected [19,20] (e.g. the case of *R. bursa* in the Pantelleria island and of *I. ricinus* in the Molise and Campania regions).

The above results prompted us to develop an ELISA test to screen sera for indirect signs of infection by *M. mitochondrii*, focusing the study on the dog; this test was based on a recombinant antigen from *M. mitochondrii*, the flagellar protein rFliD [11,18]. We emphasize that we do not expect cross-reactivity of this antigen toward tick-associated spirochetes, Rickettsiaceae or Anaplasmataceae; rather, cross-reactivity with other bacteria from the family Midichloriaceae cannot be excluded (see discussion below). Using this test we analyzed 218 dog sera collected from 16 kennels located in southern Italy (Table 2), and the sera from the 20 experimental dogs included as controls. The cut-off of the test was determined on the basis of results obtained from the sera from the control dogs, and positioned at 0.25 O.D. As shown in Table 2, the average O.D. values for IgG antibodies reacting with rFliD is above/equal to the cut-off in K1-6. In these six kennels the percentage of positive dogs ranges from 22.2% to 86.6%. In the remaining 10 kennels, the average O.D. value was below the 0.25 cut-off: dogs from four of these were all negative (K13-16), while some positive dogs were recorded in the remaining six, as indicated by the O.D. maximum value (K7-12; Table 2).

Considering the whole population of the dogs examined from the 16 kennels, seroprevalence is 26.6%, which is significantly different from the 5% seroprevalence of control dogs (Student t-test, P < 0.05). The 218 dogs from the kennels can generally be assumed to be at risk of tick bite, while the 20 experimental dogs can be assumed to have no risk of tick parasitism. Considering the above information, the results here reported (i.e. 26.6% seroprevalence for *M. mitochondrii* in dogs at risk of tick bite, and 5% in dogs at no risk) are congruent with the idea that antigens from *M. mitochondrii* (or from closely related bacteria) are inoculated into animals during the tick blood meal. As for the differences in the seroprevalence in dogs from the different kennels (Table 2), this could derive from management/sanitary differences among kennels, as well as from their geographic and environmental location, in relation with tick distribution in Italy. For example, in the rural environments of the Molise region, dense populations of *I. ricinus* have been recorded (Rinaldi L., unpublished observations); this can explain the 86.6% seroprevalence recorded in K1 (see Table 2), also considering that positivity for *M. mitochondrii* is 100% in all the life stages of *I. ricinus*, excluding adult males [5]. In addition, all of the kennels displaying an average O.D. above the cut-off (Table 2, K1-6) are located in rural areas, where tick presence has been recorded [21,22] (Rinaldi L., unpublished observations). On the other hand, the presence of ticks is more sporadic in the urban and suburban environments of Napoli, Salerno and Avellino counties, where kennels displaying lower O.D./seroprevalence values (K7-16) are located [21,22]. For only three of the kennels that were included in the serological screening we could collect, for a subsample of the dogs, whole blood for DNA extraction/PCR screening, in addition to sera samples (K1, K3 and K14; see Tables 1 and 2). In the case of K1 and K3, one PCR positive dog was detected in each kennel (K1, one out of four; K3, one out of seven; see Table 1); the O.D. values for these two PCR-positive dogs were respectively 0.67 and 0.37, i.e. both of them can be classified as seropositive for *M. mitochondrii*. Being based on a subsample, the above results do not, however, allow us to estimate any correlation between serological and PCR positivity. It is anyway interesting that the two kennels where serological positivity is higher present PCR-positive dogs (K1 and K3), while no positive dog was detected in a kennel where all of the animals are seronegative for *M. mitochondrii* (K14)*.*

The antigen used for the above ELISA screening (i.e. rFliD) is a component of the flagellum of *M. mitochondrii* from *I. ricinus*. This bacterium is rather peculiar in that it is the sole Rickettsiales so far shown to possess a flagellar structure: the well-established pathogenic Rickettsiales form the genera *Rickettsia, Ehrlichia* and *Anaplasma* do not have flagella [23]. We would thus exclude that results of the above serological screening derived from cross-reactivity with antigens from other Rickettsiales. On the other hand, *Borrelia burgdorferi* sensu lato, the main pathogen transmitted by *Ixodes* ticks, does possess immunogenic flagella. However, published results show that, in humans exposed to tick bite, a high proportion of the subjects positive to *M. mitochondrii* are negative to *B. burgdorferi* (and vice-versa), indicating the absence of immunological cross-reactivity among the FliD proteins of *Midichloria* and *Borrelia* bacteria [11]. On the other hand, cross-reactivity of rFliD could be expected toward other bacteria from the genus *Midichloria*, under the reasonable assumption that other bacteria from this genus possess the flagellar gene *fliD*.

The work reported here presents two lines of evidence that suggest that *Midichloria* bacteria circulate in mammalians: 1) direct signs of their presence, i.e. the detection of DNA gene fragments in blood samples from different animal species, that cluster in the *Midichloria* bacterial genus; 2) indirect signs of their presence, i.e. the detection of anti-*Midichloria* antibodies in dog sera. These results do not of course allow us to infer any conclusion about whether *Midichloria* bacteria replicate in mammalian hosts: ticks (or other arthropods) might simply inoculate *Midichloria* (or DNA/proteins from these bacteria), in an amount sufficient for PCR detection and to stimulate antibody production. However, considering the amount of blood in the animals here examined (e.g. horses), we believe it would be unlikely that bacterial DNA inoculated by a few ticks, and diluted into liters of blood, could then be detected by PCR, by the analysis of the DNA extracted from 100 microliters of blood, in the absence of a multiplication in the animal host. We are thus more prone to consider the possibility that *Midichloria* bacteria multiply in the mammalian host. As for the antibody response toward the rFliD *Midichloria* antigen, repeated inoculations of antigens from *Midichloria* by several ticks, as it might occur in animals, could be sufficient to stimulate an antibody response. On the other hand, the seropositivity for *Midichloria* recorded in humans parasitized by ticks is suggestive of a replication of these bacteria, considering that single ticks had generally been removed from the examined subjects, normally after a very short blood meal [11].

## Conclusions

In summary, our current work on animals, together with the previously published study on humans [11], presents overall evidence that ticks inoculate *Midichloria* bacteria into mammalian hosts during the blood meal, and that these bacteria likely multiply, inducing an antibody response. Whether inoculation of *Midichloria* bacteria could lead to the infection of the host, and whether this could determine pathological alterations, is now a main research issue. In addition, investigations on the immunology of the tick saliva [24] should now take into account the possible presence and potential immunomodulatory activity of *Midichloria* and *Midichloria*-associated molecules.

## Competing interests

The authors declare that they have no competing interests.

## Authors’ contributions

CHB, CLB and SE conceived the study and contributed to data analysis, interpretation and manuscript writing. MM, SE and EM performed the molecular and serological analyses; LR, GC and CG contributed to sample collection. DS and SU performed the phylogenetic analysis and contributed to data interpretation and manuscript writing. All authors read and approved the final version of the manuscript.
